# Electrochemical Detection of Glucose Molecules Using Laser-Induced Graphene Sensors: A Review

**DOI:** 10.3390/s21082818

**Published:** 2021-04-16

**Authors:** Jingrong Gao, Shan He, Anindya Nag

**Affiliations:** 1 College of Light Industry and Food Science, South China University of Technology, Guangzhou 510006, China; gaojingrong2116@sina.com; 2 School of Chemistry and Chemical Engineering, Guangzhou University, Guangzhou 510006, China; he0091@gmail.com; 3 Institute for NanoScale Science and Technology, College of Science and Engineering, Flinders University, 5042 Bedford Park, Australia; 4 School of Information Science and Engineering, Shandong University, Jinan 251600, China

**Keywords:** laser-induced graphene, sensors, nanomaterials, copper, glucose

## Abstract

This paper deals with recent progress in the use of laser-induced graphene sensors for the electrochemical detection of glucose molecules. The exponential increase in the exploitation of the laser induction technique to generate porous graphene from polymeric and other naturally occurring materials has provided a podium for researchers to fabricate flexible sensors with high dynamicity. These sensors have been employed largely for electrochemical applications due to their distinct advantages like high customization in their structural dimensions, enhanced characteristics and easy roll-to-roll production. These laser-induced graphene (LIG)-based sensors have been employed for a wide range of sensorial applications, including detection of ions at varying concentrations. Among the many pivotal electrochemical uses in the biomedical sector, the use of these prototypes to monitor the concentration of glucose molecules is constantly increasing due to the essentiality of the presence of these molecules at specific concentrations in the human body. This paper shows a categorical classification of the various uses of these sensors based on the type of materials involved in the fabrication of sensors. The first category constitutes examples where the electrodes have been functionalized with various forms of copper and other types of metallic nanomaterials. The second category includes other miscellaneous forms where the use of both pure and composite forms of LIG-based sensors has been shown. Finally, the paper concludes with some of the possible measures that can be taken to enhance the use of this technique to generate optimized sensing prototypes for a wider range of applications.

## 1. Introduction

The need for sensors arose at the end of the 20th century, when researchers had started using these devices for applications that directly affected human life. In the times when the involvement of sensors was not popular, the completion of certain daily activities took more time and energy, with less precision. The involvement of sensors assisted in improving the quality of life in dealing with the curbing issues. In the beginning, due to the high fabrication cost and limited supply of sensors, they were selectively chosen for certain industrial applications. The introduction of semiconducting sensors, around three decades ago, led to an increase in the range of applications to a great extent [1,2]. Investigations done on the fabrication techniques and processing materials helped to develop enhanced semiconducting sensors in terms of their respective performances. Single-crystal silicon substrates [3,4] were the most favored among the processed materials, while the microelectrochemical systems (MEMS) fabrication process [5,6] was standardized to form the prototypes. Some of the critical advantages of these types of prototypes were small size, high signal-to-noise ratio, low hysteresis and high repeatability in their responses [7]. Although semiconducting sensors served a great purpose for biomedical [8,9], industrial [10,11] and environmental [12,13] applications, there are certain drawbacks that compelled researchers to opt for an alternative paradigm. The brittle nature and the high cost of fabrication of semiconducting sensors were some of the major disadvantages that limited their widespread uses. 

Researchers then chose flexible materials to develop suitable sensors that could overcome the drawbacks of the MEMS-based sensors, in addition to certain advantages related to electrical, mechanical and thermal attributes. A range of raw materials has been considered to form flexible sensors that can operate with high efficiency [14,15]. Along with the high quality of raw materials, the wearable nature of the flexible sensors increased their applications via ubiquitous sensing [16]. The substrates and electrodes of flexible sensors have been fabricated using a range of polymers and conductive nanomaterials, respectively. Each of these materials has been chosen on the basis of the electromechanical characteristics imparted by them in the final prototype. Out of a wide range of polymers that have been utilized for devising the substrates, some of the common ones are polydimethylsiloxane (PDMS) [17,18], polyethylene terephthalate (PET) [19,20], polyimide (PI) [21,22] and poly (3,4-ethylenedioxythiophene) polystyrene sulfonate (PEDOT: PSS) [23,24]. To form the electrodes, scientists have been using nanomaterials of varied kinds for some time [25,26]. This sector includes particles in micro and nano ranges that provide exceptional qualities to the fabricated devices. Some of the primary advantages of these materials include increased surface area, high reliability, robustness, high electrical conductivity and mechanical flexibility [27,28]. The nanomaterials have been used in different forms, including powder [29,30] and particle [31,32] nature. The choice of these forms mainly depends on their capability to integrate with other associated raw materials in the sensors. Along with these two physical forms, the nanomaterials can further broadly be classified into two different types: carbon-based allotropes and metallic nanostructures. The first category comprises certain elements like carbon nanotubes (CNTs) [33,34], graphene [35,36] and graphite [37,38], while the second category includes dimensional variation like nanowires, nanorods and nanoparticles, that are formed using the d-block elements of the periodic table. Gold [39,40], silver [41,42], iron [43,44] and copper [45,46] are some of the common metals that have been used to develop these structures. Each of the structures has its unique way of embedding and imparting essential characteristics to the resultant prototypes. Although all the mentioned nanomaterials have been considerably used to form flexible sensors, we will highlight the significance of graphene in enriching the quality of the sensors to a great extent.

**Figure 1:**
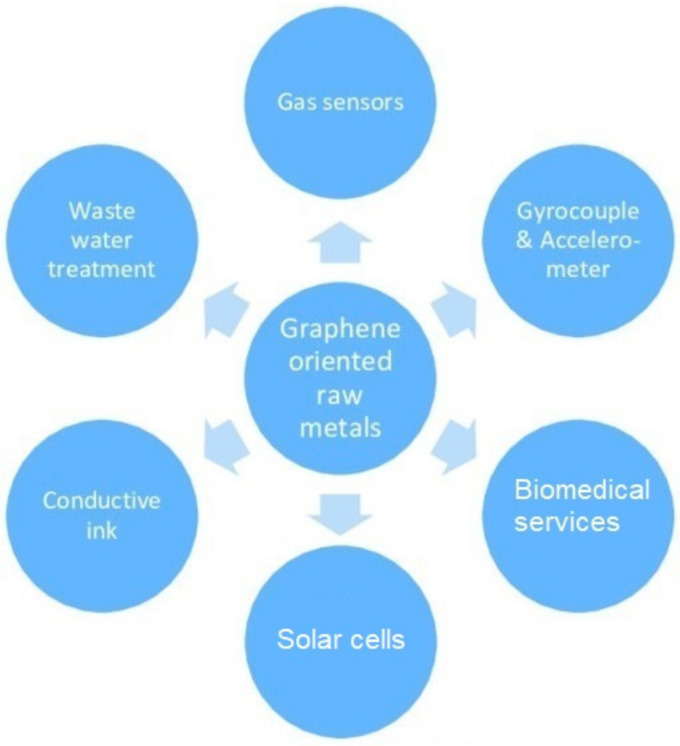
Overview of the use of graphene for different sensory applications [58]. Copyright 2019 AGH University of Science and Technology.

Followed by the rise of graphene [47], this material has been extensively used to develop sensors due to its excellent electrical, mechanical and thermal characteristics [48,49]. The presence of hexagonally arranged sp^2^-bonded carbon atoms has shown very high thermal conductivity, mobility and surface area with values 5000 Wm^−1^ K^−1^, 10,000 cm^2^ V^−1^ s^−1^ and 2630 m^2^ g^−1^, respectively [50]. Some of the other primary advantages essential for forming sensing prototypes are high Young’s modulus and high optical transparency in the visible wavelength range. Graphene has been fabricated using a wide range of methods like chemical vapor deposition (CVD) [51,52], Hummer’s method [53,54] and modified Hummer’s method [55,56]. From recent studies, it has been found that these synthesis techniques have helped to use this material to form a wide spectrum of wearable and flexible sensors [57]. Figure 1 [58] shows a pictorial representation of some of the primary uses of graphene among its diversified applications. It is seen that the utilization of graphene has been done in almost all research and academic sectors [59,60,61,62,63,64]. Although these techniques serve a great purpose, researchers have constantly been working to reduce the cost of fabrication, while maintaining the quality of the prototypes. The above-mentioned synthesis methods have certain limitations like temperature restrictions and production of toxic gases related to CVD, while the presence of defects and high processing time are associated with Hummer’s method. 

**Figure 2:**
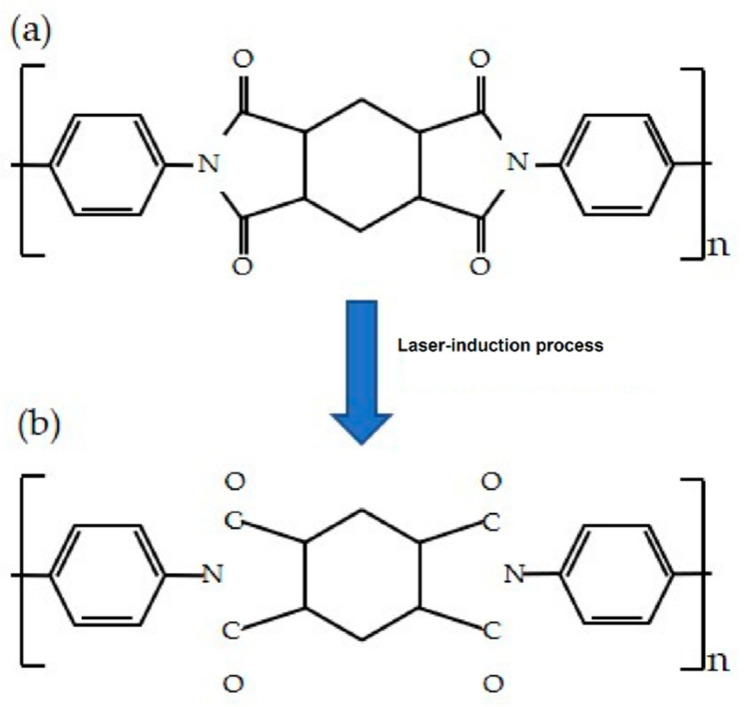
Principle of the formation of laser-induced graphene. Structure of polyimide film (**a**) before and (**b**) after the laser irradiation process [85]. Copyright 2019 MDPI.

The use of the printing process has been one of the most effective techniques in developing flexible materials [65,66]. Some of the advantages of the printing process are the low cost of the sensors, high-quality sensors in terms of sensitivity and robustness, high customization of the prototypes, and the ability to form biocompatible sensors that can be used for the detection of critical parameters physiologically and anatomically. A few of the printing processes are screen printing [67,68], inkjet printing [31,69], gravure printing [70,71], laser-ablation [72,73] and 3D printing [74,75]. Among them, laser-induction or laser-ablation process has been state-of-the-art in the field of microelectronics to design and develop sensors for different applications [76,77,78,79]. With certain advantages linked to this process, including quick fabrication, minimal requirement of post-processing steps and generation of ultra-flexible thin-films, it has been considered to develop a large number of electrochemical and strain sensors. A wide range of sectors have been covered with this technique, including material science to biomedical applications [80,81,82,83]. This methodology primarily operates on the photothermal and photochemical transition of substrates to form the desired material. The amount of precision offered by this technique is difficult to achieve in other standardized thermal approaches. This is why it is the most preferred technique in the current scenario over the wet chemical techniques to form graphene oxide (GO). Laser-induction of graphene was first reported in [84], where commercial polymers had been directly laser scribed with a CO_2_ infrared laser system to form porous graphene structures. Figure 2 [85] shows the schematic diagram of the principle of laser-induced graphene (LIG). The process involves the conversion of sp^3^ hybridized carbon atoms of the substrates to sp^2^ hybridization of those of graphene. This conversion takes place via breaking of the C-N bonds due to its weakest binding force, in addition to the formation of other unstable compounds like CO, CN, C_2_, C, CH, C_2_H_2_ and HCN. Since then, scientists have exploited a lot of materials to generate 3D graphene material. A few such materials include PI [86,87], polyetherimide (PEI) [88], phenolic resin [89,90], polystyrene [91,92] and wood [93,94]. Some of the advantages of the graphene formed with this technique include high surface area, high thermal stability and high electrical conductivity. Other experimental step-related benefits are completion of the entire process in ambient air, avoidance of requirement of solvents and single fabrication step. 

**Figure 3:**
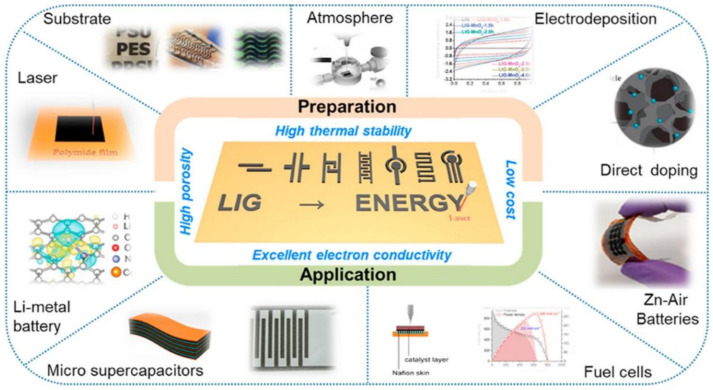
Illustration of the use of laser-induced graphene (LIG) for different applications [97]. Copyright 2020 Elsevier.

The formation and utilization of LIG to develop flexible sensors have been done on a large scale [95,96]. This induced graphene has been used for different kinds of applications, as represented in Figure 3 [97]. These prototypes operate in different mechanisms like electrochemical, strain and electrical sensing. The specifications of the induced graphene in terms of its electromechanical attributes were based on the individual applications for which they have been employed. Among them, the performance of the sensors used for electrochemical applications has been outstanding due to the high surface area, porosity and mobility of graphene. The 3D-printed graphene generated with this technique has been used in pure and composite forms to determine the concentrations of ionic molecules present in the laboratory and real-time solutions. The point-of-care devices developed with LIG helped to achieve high efficiency in terms of operating range sensitivity for the target molecules. The analytes are mostly detected as biomolecules on the labeled or label-free sensing area of the prototypes. Out of all the major ionic molecules targeted in biomedical applications, glucose has been one of the critical ones whose presence in the human body at precise concentration is essential for normal functioning. Its significance mainly lies in its being the primary constituent of energy sources. The concentration of glucose present in a normal human body ranges from 3.3 to 7.8 mol/L [98]. The reduction of glucose levels beyond this range can lead to hypoglycemia, which can lead to anxiety, fast heartbeat, dizziness, confusion and nervousness [99]. Excess glucose levels can also be harmful, as it leads to hyperglycemia, which can increase the risk of heart and kidney diseases. Thus, maintaining an optimum glucose level in the body is of utmost importance as far as health experts are concerned. 

Researchers all over the world have been trying to develop low-cost glucose sensors that can be used as point-of-care devices for quick testing purposes. Although review articles have been published before [100,101,102,103,104] related to glucose sensors, none of the papers have reviewed the use of LIG sensors for glucose detection. The novelty of this paper lies in the significance of LIG-based sensors for electrochemical detection of glucose molecules. The explanation regarding the related research work has been categorized based on the types of sensors that have been employed for sensing purposes. The mentioned examples have been grouped into two primary categories, including the ones functionalized with metallic nanomaterials and other forms. The first category has been further sub-divided into two forms, namely, the ones functionalized with copper nanomaterials and other kinds of nanomaterials. The use of copper nanomaterials for glucose detection has been done extensively due to its better catalytic activity, detection limit, high stability and operating range [105]. The catalytic activity of these copper nanomaterials had been constantly increased by varying the surface particle, size and texture of the copper particles [106]. Other types of nanomaterials used to functionalize the LIG-based sensors include gold, platinum and others. The other types of LIG-based sensors used for glucose detection include the integration of polymeric materials with the electrodes of the sensors. Each of the examples has been chosen differently to underline their importance and capability for glucose detection. 

## 2. Use of LIG-Based Sensors for Detection of Glucose Molecules 

The effectiveness of LIG in electrochemical applications has been very advantageous in comparison to other conductive elements. Some of the advantages of LIG for these criteria include high electrical conductivity, high porosity, single processing step and avoidance of any wet chemical steps [107,108]. The fabrication of these electrochemical sensors is generally carried out using a range of light-emitting sources in an ambient atmosphere. Certain features like the spatial resolution of the LIG have been optimized over time to increase the sensitivity and reduce the response time of the prototypes. These optimization processes have been carried out using light-emitting sources by varying certain parameters like pulse per index and radiation energy. The carbonization process carried out to form the LIG decides the morphologies of the designed structures. The constituents of the generated LIG have been varied in terms of its surface characteristics to broaden its usability in sensors and microfluidic fields. Among the sensorial applications, their representation for the detection of glucose molecules has been done here. The LIG has been either used in its pure form or combined with other composite materials to form the prototypes for characterization and experimentation purposes. The raw materials used to generate the LIG vary among different kinds of carbonaceous materials, thus varying the characteristics of the resultant prototypes. Table 1 [78] shows a comparison between the research works that generated LIG using different substrate materials and laser sources. It is seen that due to the variation in the choice of the source material, the quality of the induced graphene also varies, as a result of which its corresponding characteristics vary. Each of the lasers has its own advantages that assist in the fabrication process. While continuous-wave lasers are mostly used to develop finite structures of LIG, pulsed lasers are commonly used here because of easy handling and high peak powers. Ultrashort pulsed lasers are barely used to develop electrochemical sensors, as they primarily develop LIG-based sensors with very precise structures and fine edges. The differences in the intensity of the laser attributes and a variation in the precursor material create graphene with varied characteristics. Since the operation involves photo-thermal conversion, researchers are constantly trying to exploit regularly used carbonaceous objects containing double-bonded carbon atoms [109]. The discrepancy in the performance of the LIG also lies with the energy densities, which correspondingly varies with the type of laser nozzles and light sources. The pulsed and ultra-shot pulsed lasers that operate with a pulse with less than 180 fs provide an additional advantage of avoidance of drop in spatial resolution [110]. Primary morphological characteristics of porous graphene depend on other laser parameters like speed and frequency of the incident radiation, thus originating different structural rearrangements [111]. The rasterization of the laser beam over the substrates helps to gain control over the morphology via determining the host of process parameters like writing environment and amount of area for charge retention near the edges of the electrodes. One of the interesting facts related to optimized generation of LIG output is the presence of organic ligands, which not only maintains the homogeneity on the substrate but also enhances the performance of the formed electrodes. For example, while using the LIG as supercapacitors, the presence of organic ligands linking with metal cores acts as pseudo-capacitive electrodes [112]. The research works exemplified in this paper include continuous and pulsed wave lasers for synthesis purposes. The laser power primarily depends on the type of laser used to photothermally convert the substrate material. This varies certain attributes of the induced grapheme, such as porosity and conversion rate. The variations in the shapes and structural dimensions of the LIG-based sensors affect their performances in terms of efficiency, robustness and longevity.

### 2.1. Metallic Nanomaterials-Functionalized LIG-Based Glucose Sensors

The surface modification of LIG-based sensors using nanomaterials has been one of the effective steps taken for the detection of specific ionic molecules. This alternation in the surface of the electrodes helped to capture the target analyte with high specificity. The externally functionalized particles used on the prototypes bonded with the conductive metals to increase their sensitivity. A lot of nanomaterials to date have been tested to impart their attributes to the parent prototypes for different types of applications. Among the sensors used for electrochemical detection of glucose molecules, the functionalized materials mostly consist of metallic nanoparticles whose enhanced characteristics depend on their size, structure and dimensions. The choices of nanomaterials being bonded with the porous graphene have varied certain sensing parameters like linear range, limit of detection (LOD) and others. Table 2 shows a comparison of some of the common metallic nanoparticles in their pure and oxide forms that have been considered to detect the glucose molecules. It is seen that not only the type of metallic nanoparticles varies in each work, but the analytical methods also differed in accordance with the operating mechanism of the prototypes. The performance of each of the prototypes differs in terms of linear range and LOD. In extrapolation of the research works mentioned in the table, this paper classifies the use of some of the major metallic nanomaterials that have been used to detect glucose molecules. The first category explains different forms of copper nanomaterials as they have been extensively used for the detection of glucose molecule. The second class includes some efficient metals like platinum, Prussian Blue (PB), gold, nickel and cadmium. In both the categories, the fabrication process and performance of the prototypes have been shown to depict the differences between the mentioned examples.

#### 2.1.1. Functionalization Using Copper Nanomaterials

The significance of copper nanomaterials for detecting glucose molecules lies in their multiple oxidation states, low procuring cost, abundance and varied forms. Each type of nanostructured copper form integrates perfectly with the substrates to perform the electrochemical catalytic activity necessary for detecting the molecules in micro concentrations. One of the works [139] shows the use of copper nanocubes (Cu NCs), where the researchers developed flexible sensors by electroplating these elements on the LIG. The LIG formed using CO_2_ lasers was decorated using Cu NCs to achieve highly selective sensors. The average sheet resistance values in the absence and presence of Cu NCs were 15.6 Ω/cm and 19.6 Ω/cm, respectively. The chemical vapor (CV) technique used for the analysis purpose was used to detect the changes in the responses even at bent conditions of the flexible electrodes at 30°, 90° and 180°. A high sensitivity of 1643.31 µA/mm·cm^2^ was achieved for glucose concentrations with a linear range of 0.05 mm to 1 mm. The LOD of the sensors for the tested molecules is 0.05 mm. The peak voltage was obtained at 0.42 V when the glucose level measurements were done from human physiological samples like saliva, tear and sweat.

**Figure 4:**
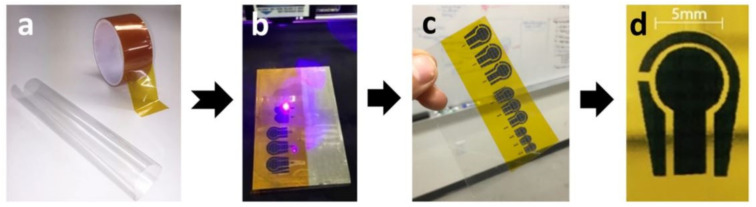
The fabrication process of the LIG-decorated sensors for the detection of glucose molecules [140]. (**a**) Raw materials included Kapton tape and PVC sheet. (**b**,**c**) The direct laser reduction of Kapton tapes to form graphene. (**d**) Formation of DREG-3 electrode platform. Copyright 2016 Springer Nature.

Another example related to the use of Cu NCs to modify the surface of LIG sensors for the detection of glucose molecules can be seen in the work done by Tehrani et al. [140]. Disposable glucose sensor strips were fabricated using direct LIG that were decorated with Cu NCs. The sensors were developed using a three-step fabrication process, which was advantageous for large-scale production purposes. Figure 4 [140] shows the fabrication steps followed to develop the sensors. The laser engraving was done on Kapon tapes that were attached to thin, transparent polyvinyl chloride (PVC) sheets. CorelDraw Graphics was used as the graphic software to develop the 3-electrode prototypes. Optimization was done on the laser power intensity, focal point and distance between the laser beam and Kapton substrates to complete the process. The formed graphene electrodes were padded with copper tapes. The connection of the sensors was further improved by including silver paste to the electrodes. The formation of LIG was followed by electrochemical deposition of nano cubic structured copper particles, done via a pulsed electrodeposition process. A pulse current intensity of 200 µA was used for 350 cycles to conduct the operation on the 3-electrode system. The sensors were tested using the CV technique for the detection of glucose molecules. The sensors showed an excellent selectivity towards the target molecules with a high sensitivity of 4532.2 µA/mm·cm^2^. The linear range of the sensors was from 25 µm to 4 mm, having a LOD of 250 nm. The reproducibility and stability of the sensors were 96.8% and 97.4%, respectively. 

**Figure 5:**
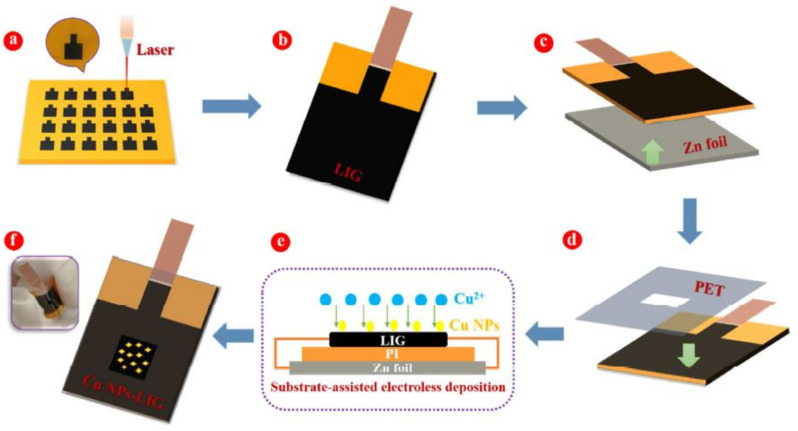
Schematic illustration of the fabrication of the Cu nanoparticles (NPs)-modified LIG-based sensors [142]. (**a**) The laser induction of the PI films was done (**a**) The LIG was formed form PI films. (**b**) A part of the films was the induction process was carried out, (**c**) it was connected to zinc films. (**d**) The whole structure was encapsulated with PET films and (**e**,**f**) dipped into copper sulfate solutions to form Cn-functionalized LIG sensors. Copyright 2020 Elsevier.

A different category of copper nanomaterials that have been used for glucose detection can be seen in the utilization of copper nanoparticles (Cu NPs) [141], where they were used to decorate porous LIG for forming non-enzymatic electrochemical glucose sensors. The electrodes were formed using a CO_2_ pulsed laser in a faster mode. The optimized power, speed, pulse per inch (PPI) and dots per inch (DPI) were 8.9 W, 9 cm/s, 1000 and 500, respectively. The formed patterns were cleaned with acetone, methanol and deionized (DI) water, following which a layer of PI tape was used to protect the electrode lines. The bonding pads of the sensors were covered with silver epoxy to protect them during the experimental process. The sensors were tested using a three-electrode system, where the working, counter and reference electrodes were formed using LIG, graphite rod and Ag/AgCl, respectively. A sensitivity and LOD of 1438.8 µA/mm·cm^2^ and 124 nm were respectively achieved using the developed Cu NPs-modified LIG-based sensors. A broad linear range was also obtained with these sensors for an applied potential of +600 mV. Another work on the use of Cu NPs for modifying the LIG-based sensors for glucose detection can be seen in [142]. A simple substrate-assisted electroless deposition (SAED) technique was used to form flexible, non-enzymatic sensors. Composites were formed using Cu NPs and LIG to increase the selectivity of the prototypes. Figure 5 [142] shows the schematic representation of the fabrication process for these Cu NPs-modified sensors. After the PI films were cleaned using alcohol and dried at 60 °C for an hour, laser scribing was done with optimized power ranging between 150 mW and 400 mW.

After the LIG was formed, the samples were cut into small pieces and attached to the same sizes of zinc foil using silver paste. Then, the samples were encapsulated with PET for electrochemical sensing purposes. The encapsulated samples were then taken for the SAED process to finalize the prototypes by removing the zinc foil and silver paste. The high-resolution transmission electron microscopic images revealed that an interplanar spacing of 0.209 nm was present between the adjacent locations of the cubic copper structures. The sensors were operated using a three-electrode system for testing different concentrations of glucose samples. The copper assists in determining the glucose concentrations by changing its oxidation surface states, which leads to the deprotonation and isomerization of the glucose molecules. A potential range of 0–0.6V was used to perform the experiments. The input voltage was increased at successive steps from 0.4 to 0.55 V under the influence of a magnetic mixer. The scan rates were increased from 8 to 200 mV/s to conduct the volumetric analysis. The prototypes were capable of determining the glucose concentrations in the presence of other similarly distributed species like uric acid and ascorbic acid. The sensitivity, response time and LOD of the sensors were found to be 495 µA mm^−1^ cm^−2^, 0.5 s, 0.39 µm, respectively. High correlation coefficients (R^2^) of 0.993 and 0.983 were achieved for anodic and cathodic current peaks, respectively. The sensors also displayed excellent stability and reproducibility of the responses. The relative standard deviation was around 2.70% for the tested glucose concentrations.

Even the oxide forms of copper have been used to detect glucose molecules, where copper oxide nanoparticles (CuO NPs) have been used to modify the surface of LIG sensors [143]. Surface engineering was done on the LIG to use the prototypes for the non-enzymatic detection of glucose molecules from the finger-pricked blood of human beings. The 3D patterned graphene was integrated with enzymes to obtain certain advantages like binder-free, highly porous and conductive carbon network. The nanoparticles helped in obtaining high catalytic efficacy by achieving a significant change in current with respect to time for the tested concentrations. The sensors were tested using the CV technique for the tested glucose molecules. The linear range of the sensors was 1 µm–5 mm, along with a response time and LOD of 0.2 s and 0.1 µm, respectively. The sensors also displayed high stability and reproducibility in the results when tested for glucose in different human body fluids like whole blood, serum, sweat and urine. The wearable nature of the sensors was also added to the induced graphene by transferring it to commercial Scotch brand tapes. Table 3 shows the summary of the performances of the copper nanomaterials-functionalized LIG-based sensors for the detection of glucose molecules.

#### 2.1.2. Functionalization Using Other Nanomaterials

**Figure 6:**
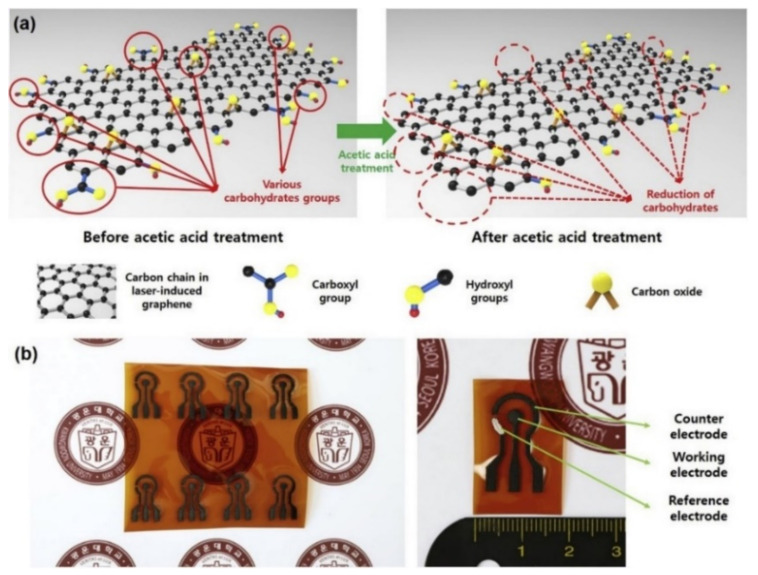
(**a**) Surface-modification of the sensors. (**b**) Chemical vapor (CV) technique was used to test the prototypes for glucose measurement [145]. Copyright 2020 Elsevier.

Apart from the copper nanomaterials, other common metallic elements have also been successfully used to detect glucose molecules with high efficiency. Yoon et al. [145] elucidate one of the works where the use of platinum nanoparticles (Pt NPs) modified LIG-based electrochemical sensors for sweat glucose detection. The sensors were formed using a PI film, having a thickness of 125 microns. The power, wavelength and scan rate of the CO_2_ IR lasers were 9.6 W, 10.6 µm and 150 mm/s. The pore size and the surface area of the LIG electrodes were <9 nm and ∼349 m^2^/g, respectively. Surface modification of the LIG-based sensors was done using acetic acid to increase their sensitivity. The acid treatment of the sensing area was done using a facile and practicable dipping technique. Figure 6 [145] shows the schematic diagram of the modification process of the LIG electrodes. The surface modification assisted in the enhancement of the conductivity of the sensors by decreasing the carbohydrate functional groups. This decrease subsequently led to an increase in the ratio of carbon-carbon bonds. Followed by the modification of the surface, the working electrode was formed through the even dispersion of Pt NPs via electrodeposition technique. Then, the conductive composites were immobilized with chitosan–glucose oxidase to form the prototypes. The sensors were tested using the cyclic voltammetry (CV) technique. The sensors exhibited high sensitivity and low LOD of 4.622 µA/mm and 300 mm, respectively. The signal-to-noise ratio of the sensors was 3, while the dynamic linear range extended up to 2.1 mm. The standard deviation of the responses of the sensors when the samples were tested for 16 days was from 0.36 µA to 0.66 µA. Another work showing the use of Pt-functionalized LIG for detection of glucose molecules can be seen in [146], where wearable patches were utilized for sensing purposes at ultra-low detection limits. CO_2_ lasers were used to develop LIG-based electrodes that showed high stability, excellent mechanical flexibility and electrical conductivity. The selectivity was increased by electrodepositing Pt nanoparticles, which increased the sensitivity and detection range of glucose molecules from sweat.

Lei et al. [147] showed another interesting work related to the use of chemically-based LIG sensors for the detection of glucose molecules. The sensors were developed through the doping of the nitrogen element that was obtained from a lignin-based precursor. The Blade-coating technique was used to process the precursor films to form homogeneous solutions. CO_2_ lasers were used to complete the process with the desired size and structure. The doping was done with LIG to form binder-free, highly conductive, hierarchical sensors. The electrodes of the prototypes were further functionalized by MXene and Prussian Blue (PB) nanoparticles using a spray-coating process. The prototypes were assembled via immobilization of the catalytic enzymes to access the composite-based electrodes. The high electrochemical activity was displayed by these sensors as a result of high interconnectivity between the carbon networks, causing an enhanced heterogeneous transfer rate of electrons. The high porosity and enriched active edge plane sites of these LIG sensors helped in the detection of multiple compounds like glucose, lactate and alcohol. Chronoamperometry (CA) and CV techniques were used to determine the glucose molecules with a sensitivity of 49.2 µA mm^−1^ cm^−2^. The glucose molecules were tested from artificial sweat with an operating range from 10 µm to 5.3 mm. This was done by immobilizing glucose oxidase to induce the selectivity of the sensors. The LOD and the signal-to-noise ratio (SNR) of 0.3 µm and 3, were respectively found.

Other than platinum and PB, gold and silver have also been used as metallic elements to functionalize the surface of LIG-based sensors for glucose detection. [148] showed the use of gold nanoparticles (Au NPs) and silver nanowires (Ag NWs) to develop stretchable composite-based hybrid sensors. The sensors displayed high, uniform electrical conductivity both under the presence and in the absence of mechanical deformations. The sensing surfaces of the prototypes were modified using Pt and Au nanoparticles to enhance the electrochemical performance of the devices. AutoCAD software was used to design the electrodes, which were fabricated using CO_2_-based lasers. Commercial PI films were washed and laser-induced to form graphene. These LIG samples were dipped into Ag NWs dispersions to have a thin-film coating over them. Then, another layer of PDMS film was coated over each Ag NWs-modified PI film. The samples were then cured at 90 °C for an hour and then peeled off from the PI templates. Then, Ag NWs were etched with a nitric acid solution, followed by a coating of liquid PDMS mixture on top of the electrodes. The samples were then functionalized with Pt and Au nanoparticles using an electrodeposition process through a potentiostat configuration. Further functionalization of the samples was done with glucose oxidase by immobilizing them on the surface area. This was carried out by casting a mixture containing glucose oxidase and chitosan on the sensing surface. The samples were tested using a three-electrode sensing technique, with the addition of Nafion/ethanol solution on these modified LIG working electrodes. The linearity and LOD of the sensors were 0.99 and 5 µm, respectively. The detectable glucose range was from 0 to 1.1 mm, where glucose was measured in sweat using these wearable prototypes. The sensors also showed a good response in terms of linearity for pH measurements done between 4 and 7. Li et al. [149] showed the conjugated use of metals and semiconducting elements to develop and utilize the hybridized LIG sensors for the detection of glucose molecules. The sensors were fabricated using nickel (Ni) electrocatalyst and cadmium sulfide (CdS) semiconductor compounds on indium-tin-oxide glass substrates. A metal-complex produced using nickel and cadmium was formed with a mass ratio of 0.7:1. Polymer pallets were also added to this mixture to obtain a homogenous metal ion-containing polyethersulfone solution. The drop-casting process was done on ITO glass substrates, followed by heating them at 80 °C for 2 h. In situ, synchronous, single-step fabrication process was carried out to develop the prototypes. The laser-induction process was used to carry out the solid phase transition process for the uniform dispersion of the nickel catalysts. The power and the wavelength of the CO_2_ infrared lasers were 6.8 W and 10.6 µm, respectively. The rest of the laser parameters, such as the z-distance, DPI and the scan rate, had values of 52 mm, 1200 and 166 mm/s, respectively. The developed prototypes were tested using CV and electrochemical impedance spectroscopy (EIS) techniques to determine their responses. The sensors displayed excellent photo-electrocatalytic activity towards the target molecules. A LOD of 0.4 µm was achieved, along with a standard deviation of 5.5% at a glucose concentration of 1 mm. The sensors also displayed good stability, high reproducibility and selectivity towards the glucose molecules. Table 4 depicts a summary of the performances of the LIG-based glucose sensors that have been functionalized with other nanomaterials.

### 2.2. Other Forms of LIG-Based Glucose Sensors

The second category of glucose sensors consists of the ones that are not functionalized using metallic nanomaterials. This sub-class includes miscellaneous sensing prototypes, which consist of both pure and composite forms. These prototypes have also been able to perform successfully, thus validating the choices of materials used to fabricate them. The characterization and experimentation of these prototypes have shown that a diversified range of sensors can be considered to detect glucose over a wide range of concentrations. The working mechanism of some of the prototypes is based on effects like the quantum confinement effect [151], which helped to enhance the electrochemical properties of the LIG-based devices. As an example, [152] shows the use of unfunctionalized LIG-based sensors for the detection of glucose molecules. 2D direct laser writing technique was used to form LIG microstructures from commercial PI sheets. A continuous-wave 532 nm diode-pumped solid-state Nd: YAG laser was used for the laser-ablation purpose. The fabrication process was divided into two stages, one with the surface of PI substrates on the motorized stage with the output being subsequently sent to the web camera. Certain laser parameters like power and scanning speed were optimized during the fabrication process. The power was varied between 60 and 120 mW, whereas the speed was varied from 0.25 to 1.1 mm/s. A power meter was used to monitor the values of these parameters. CV technique was used to determine the response with Ag/AgCl as a reference electrode and glass-like carbon as an auxiliary electrode. The concentration of the reference electrode was 3.5 M KCl. The potential range was between –0.2 and 1 V, with a constant scan rate of 50 mV/s. During the testing period, the glucose solutions of the tested samples were added from the background. The high electrochemical activity was displayed towards the non-enzymatic glucose detection using the CV technique. The LOD and the sensitivity for the first linear range (0.0001–0.001 mmol L^−1^) are 0.564 µm and 294.6 µA µm^−1^ cm^−2^, respectively. For the second linear range (0.001–0.01 mmol L^−1^), the values were 6.31 µm and 252.3 µA µm^−1^ cm^−2^, respectively.

Another work that is categorized under this sub-class can be seen in [81], where PDMS-based microfluidic biofuel cells were developed using LIG bioelectrodes. LIG obtained from PI sheets was integrated into a microfluidic device that was formed using conventional soft lithography on PDMS. The power and speed of the CO_2_ lasers were optimized during the irradiation process. The PI sheets were cleaned using isopropyl alcohol and DI water before using them for irradiation purposes. The final specifications for the synthesis process were an input power and speed of 1.5 W and 0.624 cm/s, respectively. The LIG was immersed inside a linker solution to utilize 1-ethyl-3-(3-dimethyl aminopropyl) carbodiimide to attach the glucose oxidase and laccase on the sensing surfaces. The solutions formed using these enzymes were drop-cast on the irradiated surface and then left for 2 h at room temperature for the immobilization process. The microfluidic cells were formed by cutting PET sheets with a Y-shaped microchannel. The designs for these molds were formed using CorelDraw software. Then, the liquid PDMS was cast and cured on the templates, followed by peeling them off to create microchannel structures. The dimensions of the Y-shaped microchannel included a length, width and height of 25 mm, 4 mm and 0.2 mm, respectively. The power density and fluid rate of the microfluidic cell were 13 µW/cm^2^ and 200 µL/min, respectively. Electrochemical redox reactions and polarization processes in the microfluidic ambiance assisted in obtaining excellent performance from the developed system. The linear range of the sensors was 10–40 mm, where the current showed a linear increase with respect to voltage.

**Figure 7:**
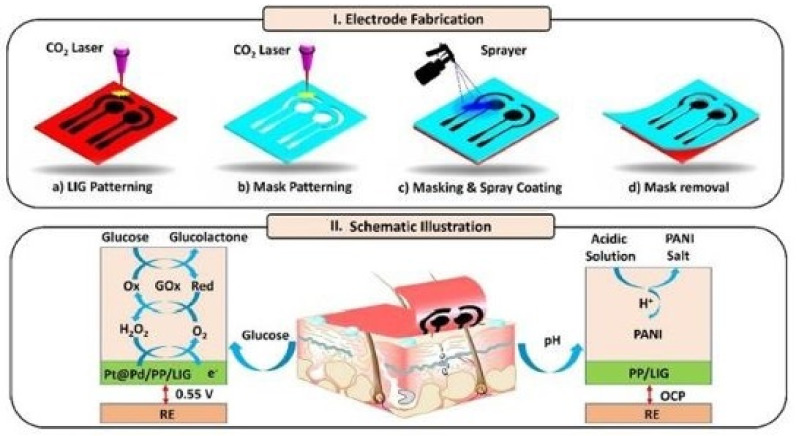
Schematic representation of the (**I**) fabrication of the sensors and (**II**) working mechanism of the prototypes [153]. Copyright 2020 Elsevier.

Zahed et al. [153] showed the fabrication of highly flexible, conductive glucose sensors through the use of PEDOT: PSS on the 3D porous LIG networks. The spray-coating process was used to include PEDOT: PSS on the LIG surface to enhance the selectivity and robustness of the prototypes. Figure 7 [153] shows the schematic diagram of the fabrication process and operating principle of the prototypes. The PI sheets with a thickness of 100 µm were laser-ablated using optimized power and a speed with values of 17 W and 6 cm/s, respectively. PEDOT: PSS was then mixed with ethylene glycol at different concentrations and spray-coated on the LIG sensor over a mask. The mask was patterned using a laser and attached with adhesive polymer tapes prior to the spraying process. The coating process was carried out in the presence of N_2_ gas flow for a duration of 30 s. Finally, the masks were peeled off and used as the working electrode in a three-electrode system for measuring glucose molecules. Glucose oxidase was used to form dispersions, which were drop-cast on the sensing area for experimental purposes. The sensors were used for the detection of glucose molecules whose responses were measured through the amperometric technique. The prototypes exhibited a high sensitivity but a low LOD of 247.3 µA mm^−1^ cm^−2^ and 3 µm, respectively. A wide linear range of concentrations between 10 µm and 9.2 mm was tested with high selectivity. The sensors also showed an excellent response in terms of sensitivity towards the pH measurements done with these sensors. The wearable nature of the prototypes was highlighted by using them to determine human perspiration during physical exercise.

**Figure 8:**
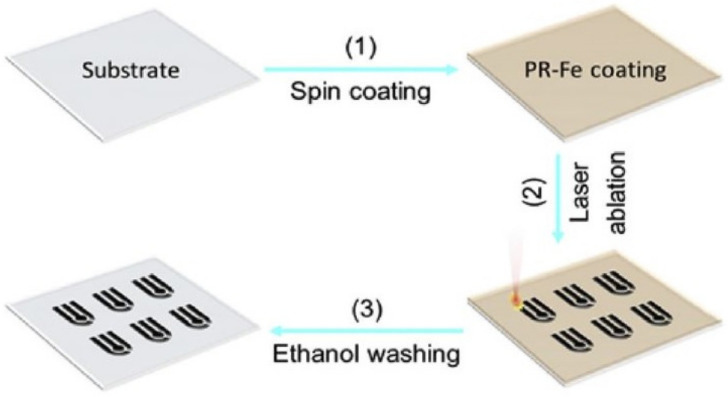
Schematic diagram of the fabrication process of the LIG patterns using visible light 305 nm semiconductor lasers [89]. Copyright 2018 Elsevier.

Zhang et al. [89] highlighted a different side of fabricating LIG from the phenolic resin using visible light. Scalable construction of induced graphene was carried out on diversified substrates, including polymer films, glass slides, metal foils, ceramic plates and plant leaves. 305 nm semiconductor lasers were utilized to form graphene, having certain attributes like porous structure, low resistance, excellent mechanical properties. Figure 8 [89] shows the schematic diagram of the fabrication process of the LIG patterns. PET films along with other substrates were used to hold the PR solution, which was drop-casted and controlled by adhesive tapes. The scan rate, power and laser wavelength values were optimized to 120 mm/s, 5 W and 10.6 µm, respectively, during the engraving process. Interdigitated electrodes were formed, which were operated as electrochemical and electrical sensors by detecting glucose molecules and as supercapacitors, respectively. Cyclic voltammetry and glavanostatic cyclic tests were carried to determine the changes in the response of the prototypes. The glucose concentrations were varied between 0 and 10 mm to determine the change in current with respect to potential at a scan rate of 100 mV/s.

Bhaiyaa et al. [154] showed the development of a miniaturized electro-chemi-luminescence (ECL) platform for multifunctional biosensing applications, which included the detection of hydrogen peroxide and glucose molecules. Open bipolar electrodes were formed using LIG that had certain properties like high selectivity, low-cost fabrication and excellent reproducibility in the responses. Followed by a single-step fabrication method, the ECL signals were sensed using an android smartphone with required voltage values. The design of the electrodes was drawn in CorelDraw software before being produced over PI sheets. Around 50% intensity of both power and speed was chosen to be the optimized values for forming the driving and bipolar electrodes. Then, immobilization of glucose oxidase was done over the bipolar electrodes, followed by placing these electrodes with an integrating 3D printed device holder. The sensors exhibited high linearity with a low detection limit of 0.138 µm. The prototypes had the potential for point-of-care testing for biomedical and environmental applications. The recovery rate was above 100% for the clinical testing done with randomly taken blood samples. The concentration of the blood sugar obtained from the samples was found to be 5.9 mm. Table 5 shows a summary of the examples showcased under this sub-section in terms of processed materials, fabrication technique, sensor attributes and detection technique.

## 3. Future Scope

Although a considerable amount of work has been done on using LIG-based sensors for glucose detection, there are still some loopholes that need to be dealt with in the current era. Certain characteristics of the induced graphene-like high porosity, high electrical conductivity, easy fabrication process and easy customization have allowed researchers to use this material for a lot of electrochemical applications. The electrical, mechanical, thermal, optical and magnetic attributes of graphene have been exploited in pure or composite forms to develop the sensing prototypes. Table 6 shows a summary of some of the above-mentioned examples that include the functionalization of the LIG surface with different materials. It is seen that each of the additive elements has allowed the enhancement of the performance of the prototypes in terms of their sensing parameters. One of the future works to be noted in this area is the further exploration of the involvement of metallic and semiconducting elements for functionalization purposes. Doping the sensing surface with s and p-block elements can further increase the electron mobility of the prototypes. The inclusion of metal oxides and nanocrystals would assist in diversifying the applications of LIG-based sensors [155]. Even though the consideration of different types of laser sources, as mentioned in Table 1, does provide varied products, this concept is still considerably new, so no standardization has yet been provided about the fabrication process. 

The wetting properties of LIG are another area that requires further work. Intriguing cases have been presented [156] where the LIG formed from PI film has both hydrophobic and hydrophilic nature due to the variation of contact angle. This mechanism has to be further researched to provide both the properties when the prototypes are to be used for electrochemical applications. The fabrication of this 3D porous graphene can be further extended by performing thermal oxidation of carbon-sourced materials. Instead of using PI or other polymers as precursors, materials like cotton and wood can help to produce biocompatible electronics from naturally occurring products. In terms of modification of the LIG, other types of nanomaterials should also be ventured, which would increase the selectivity of the sensors. Multifunctional sensing prototypes using LIG showed to be more encouraged to reduce the cost of fabrication of the sensor, while maintaining the overall sensitivity and accuracy of the response.

One of the ways to do this for electrochemical applications is to induce selectivity for each sensing prototype among an array of sensors. This would not only help in fabricating efficient systems for real-time scenarios but would also reduce the electronic waste thus generated. The fine control of composition, conductivity and structural dimensions of the LIG would help expand the applications to broader fields. Further optimization in the electrocatalytic activity can still be done to obtain enhanced prototypes. The integration of the LIG-based sensors with signal-conditioning circuits to realize actual functional devices would be state-of-the-art for commercialization. The addition of wireless communication protocols to these devices would further enhance their functionality, especially when considered as wearable sensors. The choice of the protocol can be based on the working mechanism and specifications of the targeted crowd.

In terms of market survey, there is an estimation for the exponential increase in the amount of use of graphene in the upcoming years. This use of graphene has been estimated to be in different forms like oxides, nano-platelets and quantum dots, which can exploit the high surface area of this material. The applications also include different sectors like automotive, composite, electronics, adhesives, 3D printing, sensors, supercapacitors and energy-harvesting devices [157]. The revenue is said to be around 2 billion USD in 2020, which is excepted to increase at a compound annual growth rate of 38.7% in the upcoming years [158,159]. Out of the various genres in which the use of graphene would be ventured, LIG is one new area that would help the researchers to synthesize and utilize graphene in a very short time. This process can easily prevail over other common methods like chemical vapor deposition, mechanical exfoliation and Hummer’s method in terms of the quality of generated graphene [160]. Since the quality of induced graphene is very high, the sensitivity would also be high for the targeted electrochemical sensing applications.

## 4. Conclusions

The paper presents a comprehensive review done on the use of LIG-based sensors for the electrochemical detection of glucose molecules. The laser irradiation or laser scribing of polymer films has helped to form 3D porous graphene. This photo-thermally induced graphene has been either used in pure or in composite forms to form prototypes for glucose detection. The composite forms include the functionalization done with different types of nanomaterials. With the high performance of the LIG-based sensors for detecting glucose in varying concentrations, it serves as a podium to form wearable sensors for detecting biochemical ions. The capability of LIG to perform in controlled environments has heightened the possibility of forming point-of-care devices for ubiquitous sensing applications. With further improvement in the quality of the LIG-based sensors in terms of robustness and longevity, these prototypes can become a cornerstone for a wide range of electrochemical applications. 

## Figures and Tables

**Table 1:** Comparison of the power source and raw materials for the generation of different laser-induced graphene (LIG) outputs [78]. Copyright 2018 Taylor & Francis Group.

Table	Light Sources	Power/Energy Density	Material	Characteristics	Ref.
Continuous-wave laser	CO_2_ laser	5.4 W	Polyimide	Increase in power decreased oxygen and nitrogen content in LIG to <3%	[84]
	CO_2_ laser	2–60 W/cm^2^	Single-layered Graphene	Obvious thermal effect	[113]
	Optically pumped solid-state laser	5 W	CH_4_ and H_2_	Rapid single-step fabrication of graphene patterns	[114]
	CO_2_ laser	27 W	Polyimide sheet	Organic ligands assisted in forming a homogenous cover.	[115]
	CO_2_ laser	138.4 mJ/cm^2^	Silicon carbide	Graphene layers appear like small islands, spreading over the substrate surface.	[116]
	CO_2_ laser	26.5 W	Graphene oxide	Low repetition rates increase the pulse-to-pulse distance, leaving graphene oxide between pulse spots.	[117]
Pulsed laser	Nd: YAG/5 ns	200 mJ/cm^2^	Single-layered Graphene	Produce visible damage	[118]
	Nd: YAG/7 ns	40 mJ/cm^2^	MLG	Local profile transformation	[119]
	248 nm/25 ns	1.2 J/cm^2^	Silicon carbide	Fabricated the graphene patterns on silicon carbide	[120]
	248 nm/5 ns	120 mJ/cm^2^	Graphene oxide	An effective approach for photo-reduction with minimal defects	[121]
	10–6 µm/14 µs	2.4–5.4 W	Polyimide (PI)/Wood	One-step preparation of porous graphene	[94]
	1–06 µm/10 ns	3.6–5.1 W	PI	Forming a unique honeycomb porous graphene	[122]
Ultrashort pulse laser	1064 nm/ps	100 W	Multi-layered Graphene	Precise control thinning graphene layers	[123]
	Eolite/30 ps	15 µJ	Single-layered Graphene	One-step induction	[124]
	Ti: Sa/130 fs	150–320 mJ/cm^2^	Single-layered Graphene	Induction threshold measurement	[125]
	Ti: Sa/50 fs	3 TW/cm^2^	Multi-layered Graphene	Single-shot damage threshold	[126]
	−/550 fs	100–500 nJ	Single-layered Graphene	Nanometer-scale patterning	[127]
	Ti: Sa/100 fs	47–968 mJ/cm^2^	Graphene oxide	Little or no graphitization	[121]

**Table 2:** Comparison between some of the common metallic nanomaterials that have been used for functionalization for the detection of glucose molecules.

Sensing Materials	Analytical Method	Linear Range (µm)	Limit of Detection (µm)	Ref.
Gold nanoparticles, Multi-Walled Carbon Nanotubes	Cyclic voltammetry	0.1–10	0.03	[128]
Polyvinyl acetate, graphene oxide, copper	Cyclic voltammetry	550–4400	53	[129]
Chitosan/Silver nanoparticles	Calorimetry	5–200	0.1	[130]
Platinum, manganese oxide	Calorimetry	5–500	0.18	[131]
Mucilage, silver nanoparticles	Cyclic voltammetry	10–2200	10	[132]
Graphene quantum dots, silver nanoparticles	Calorimetry	0.5–400	0.17	[133]
Glassy carbon electrode, titanium dioxide, polyaniline	Electrochemistry and chronoamperometry	20–6000	18	[134]
Zinc cobaltite, indium tin oxide	Cyclic voltammetry and chrono-amperometric	10–290	36.9	[135]
Graphene oxide/silver nanoparticles	Electrochemistry	2000–12,000	310	[136]
Cerium oxide nanoparticles	Fluorimetry	10–200	8.9	[137]
Polyethylenimine /Silver nanocubes	Fluorimetry	10–1000	0.8	[138]

**Table 3:** Summary of the performances of the copper nanomaterials-functionalized LIG-based sensors.

Materials	Functionalization Technique	Characteristics	Analysis Method	Ref.
Graphene, copper nanocubes, Polyimide	Electroplating	Avg. resistance value: 15.6 Ω/cm	Cyclic voltammetry	[139]
		Sensitivity: 1643.31 µA/mm·cm^2^		
		Linear range: 0.05 mm–1 m		
		Limit of detection: 0.05 mm		
Graphene, copper nanocubes, polyvinyl chloride	Electrodeposition	Sensitivity: 1643.31 µA/mm·cm^2^	Cyclic voltammetry	[140]
		Linear range: 25 µm–4 mm		
		Limit of detection: 250 nm		
		Reproducibility: 96.8%		
		Stability: 97.4%		
Graphene, copper nanoparticles, Polyimide	Chrono-potentiometry	Sensitivity: 1438.8 μA/mm·cm^2^	Cyclic voltammetry	[141]
		Limit of detection: 124 nm		
Graphene, copper nanoparticles, Zinc foil, polyethylene terephthalate (PET)	Substrate-assisted electroless deposition	Sensitivity: 495 μA/mm·cm^2^	Cyclic voltammetry	[142]
		Limit of detection: 0.39 µm		
		Response time: <0.5 s		
Graphene, copper oxide nanoparticles, commercial scotch brand tape	3D patterning	Linear range: 1 µm–5 µm	Cyclic voltammetry	[143]
		Limit of detection: 0.1 µm		
		Response time: <0.2 s		
Graphene, copper oxide,	Electrodeposition	Sensitivity: 1321.54 μAL/mmol·cm^2^	Cyclic voltammetry	[144]
		Reproducibility: 5.47%		

**Table 4:** Summary of the performances of the copper nanomaterials-functionalized LIG-based sensors.

Materials	Functionalization Technique	Characteristics	Analysis Method	Ref.
Graphene, platinum nanoparticles	Electrodeposition	Sensitivity: 4.622 µA/mm	Electrochemical impedance spectroscopy	[145]
		Signal to noise ratio: 3		
		Linear range: 300 nm–2.1 mm		
		Limit of detection: 300 nm		
Graphene, MXene, Prussian blue	Spray-coating	Sensitivity: 212.5 µA/mm·cm^2^	Chrono-amperometry and cyclic voltammetry	[147]
		Linear range: 0–10 mm		
		Limit of detection: 0.3 µm		
Graphene, platinum nanoparticles, gold nanoparticles	Drop-casting and electrodeposition	Detection range: −0–1.1 mm	Amperometry and cyclic voltammetry	[148]
		Linearity: 0.99		
		Limit of detection: 5 µm		
Graphene, Cadmium sulfide particles, Nickel nanoparticles	Drop-casting	High stability, reproducibility and selectivity	Electrochemical impedance spectroscopy and cyclic voltammetry	[149]
		Limit of detection: 0.4 µm		
Graphene, polydimethylsiloxane (PDMS), platinum nanoparticles, gold nanoparticles	Electrodeposition	Sensitivity: 865.8 µA/mm·cm^2^	Cyclic voltammetry and amperometry	[150]
		Limit of detection: 75 nm		

**Table 5:** Summary of the performances of the un-functionalized LIG-based sensors for glucose detection.

Materials	Fabrication Technique	Characteristics	Analysis Method	Ref.
Graphene, PDMS, Kapton	Laser induction, soft lithography	Linear range: 10–40 mm	Cyclic voltammetry and Energy dispersive spectroscopy	[81]
		Power density: 13 µW/cm^2^		
Graphene, Polyimide	Laser induction	Sensitivity: 252.3 µA µm^−1^ cm^−2^	Cyclic voltammetry	[152]
		Linear range: 10–40 mm		
		Limit of detection: 0.564 µm (0–1 µm) and 6.31 µm (1–10 µm)		
		Detection range: 0–10 µm		
Graphene, poly (3,4-ethylenedioxythiophene) polystyrene sulfonate (PEDOT): PSS, Polyaniline	Spray-coating	Sensitivity: 247.3 µA µm^−1^ cm^−2^	Cyclic voltammetry and Electrochemical impedance spectroscopy	[153]
		Linear range: 10 µm–9.2 mm		
		Limit of detection: 3 µm		
Graphene, PET, glass slides, metal foils, ceramic plates	Drop-casting	Linear range: 0.2–10 mm	Cyclic voltammetry	[89]
		Detection range: 0–10 mm		
Graphene, glucose oxidase, polyimide	Drop-casting	Linear range: 1–100 µm	Luminescence	[154]
		Detection range: 0.138 µm		
		Linearity (R^2^): 0.9449		

**Table 6:** Summary of the performance of the LIG-based sensors in terms of different sensing parameters.

Processed Materials	Sensitivity	Linear Range	Limit of Detection	Ref.
LIG, copper nanocubes	1643.31 μA/mm·cm^2^	0.05 mm–1 mm	0.05 mm	[139]
LIG, copper nanocubes	4532.2 μA/mm·cm^2^	25 µm–4 mm	250 nm	[140]
LIG, copper nanoparticles	1438.8A μ/mm·cm^2^	0.124–9.653 M, 1–4 mm	124 nm	[141]
LIG, Copper oxide nanoparticles		1 μm–5 mm	0.1 μm	[143]
LIG, Platinum nanoparticles	4.622 μA/mm	0–2.1 mm	<300 nm	[145]
LIG, Prussian Blue	49.2 µA mm^−1^ cm^−2^	10 µm–5.3 mm	0.3 µm	[147]
LIG, Gold nanoparticles	6.4 μA/mm·cm^2^	0–1.1 mm	5 µm	[148]
LIG, Nickel, Cadmium Sulfide		Correlation coefficient: 0.9976	0.4 µm	[149]
LIG	294.6 µA µm^−1^ cm^−2^, 252.3 µA µm^−1^ cm^−2^	0.0001–0.001 mmol L^−1^, 0.001–0.01 mmol L^−1^	0.564 µm, 6.31 µm	[152]
LIG, PEDOT: PSS	247.3 μA mm^−1^ cm^−2^	10 μm−9.2 mm	3 μm	[153]

## Data Availability

Not applicable.
